# Diverse Temperate Bacteriophage Carriage in *Clostridium difficile* 027 Strains

**DOI:** 10.1371/journal.pone.0037263

**Published:** 2012-05-18

**Authors:** Janet Y. Nale, Jinyu Shan, Peter T. Hickenbotham, Warren N. Fawley, Mark H. Wilcox, Martha R. J. Clokie

**Affiliations:** 1 Department of Infection, Immunity and Inflammation, University of Leicester, Leicester, England, United Kingdom; 2 Department of Microbiology, Old Medical School, Leeds General Infirmary, Leeds Teaching Hospitals Trust, Leeds, United Kingdom; 3 University of Leeds, Leeds, West Yorkshire, United Kingdom; Institute Pasteur, France

## Abstract

**Background:**

The hypervirulent *Clostridium difficile* ribotype 027 can be classified into subtypes, but it unknown if these differ in terms of severity of *C. difficile* infection (CDI). Genomic studies of *C. difficile* 027 strains have established that they are rich in mobile genetic elements including prophages. This study combined physiological studies, electron microscopy analysis and molecular biology to determine the potential role of temperate bacteriophages in disease and diversity of *C. difficile* 027.

**Methodology/Principal Findings:**

We induced prophages from 91 clinical *C. difficile* 027 isolates and used transmission electron microscopy and pulsed-field gel electrophoresis to characterise the bacteriophages present. We established a correlation between phage morphology and subtype. Morphologically distinct tailed bacteriophages belonging to *Myoviridae* and *Siphoviridae* were identified in 63 and three isolates, respectively. Dual phage carriage was observed in four isolates. In addition, there were inducible phage tail-like particles (PT-LPs) in all isolates. The capacity of two antibiotics mitomycin C and norfloxacin to induce prophages was compared and it was shown that they induced specific prophages from *C. difficile* isolates. A PCR assay targeting the capsid gene of the myoviruses was designed to examine molecular diversity of *C. difficile* myoviruses. Phylogenetic analysis of the capsid gene sequences from eight ribotypes showed that all sequences found in the ribotype 027 isolates were identical and distinct from other *C. difficile* ribotypes and other bacteria species.

**Conclusion/Significance:**

A diverse set of temperate bacteriophages are associated with *C. difficile* 027. The observed correlation between phage carriage and the subtypes suggests that temperate bacteriophages contribute to the diversity of *C. difficile* 027 and may play a role in severity of disease associated with this ribotype. The capsid gene can be used as a tool to identify *C. difficile* myoviruses present within bacterial genomes.

## Introduction

*Clostridium difficile* is a nosocomial pathogen responsible for gut inflammation, with resultant diarrhoea or pseudomembranous colitis. Infection caused by hypervirulent strains such as *C. difficile* ribotype 027 is a serious global challenge [1], [2]. A deletion in the *tcdC* gene of *C. difficile* ribotype 027 is thought to result in uncontrolled toxin production [3]. Furthermore, 027 strains are often fluoroquinolone resistant and sporulate readily, both of which may contribute to the success of the pathogen [4], [5]. A recent report showed that *C. difficile* 027 isolates could be divided into 23 and 5 subgroups using multiple-locus variable- number tandem repeat analysis (MLVA) and pulsed-field gel electrophoresis (PFGE), respectively [6]. These subtypes were associated with variable disease severity. It is important to establish factors that may contribute to the diversity and success of this hypervirulent ribotype and consequently to the epidemic potential of new strains.

Temperate bacteriophages have been shown to play several key roles in the evolution of pathogenic bacteria either by supplementing or modifying toxin production [7]. Studies have shown that phage infection of toxigenic *C. difficile* strains can cause an increase in toxin production [8], [9], [10]. Similarly, reports on *Clostridium perfringens* have shown that there may be a link between phage carriage and sporulation [11]. Several temperate bacteriophages have been isolated from *C. difficile* using mitomycin C as an inducing agent [12], [13], [14]. Alternative antibiotics such as norfloxacin have been shown to be highly effective as a prophage inducing agent in *E. coli* [15]. Although few studies have compared the efficacy of multiple agents to induce prophages in bacteria, clear differences in *E. coli* phage induction were seen using different inducing agents [16].

Five temperate *C. difficile* phages have been fully sequenced and annotated. Three belong to the *Myoviridae* (phiC2, phiCD119, and phiCD27) and two to the *Siphoviridae* (phiCD6356 and phiCD38-2) [9], [17], [18], [19], [20]. In addition, prophages have been identified in most *C. difficile* strains sequenced to date [17], [21]. Although temperate myovirus genes such as those that encode for the capsid, portal and holin proteins have been shown to share nucleotide homology, they have not previously been used in order to determine phylogenetic relationships within *C. difficile* bacteriophages.

Recent genomic analysis has identified mobile genetic elements including prophages in 22 of 25 sequenced *C. difficile* 027 strains, but the bacteriophage content was not characterised further [21]. In another study, when a *C. difficile* 027 outbreak strain was investigated, only single bacteriophage morphology was observed [14]. No previous studies have examined bacteriophage carriage within *C. difficile* ribotype 027 subgroups. To determine if bacteriophages are contributing to the diversity within *C. difficile* 027, the range of temperate bacteriophage types associated with ribotype subgroups must first be established. We aimed to isolate and characterise temperate bacteriophages from clinical *C. difficile* 027 subtype isolates, to determine if phage carriage correlates with subtype, and to design a molecular maker for the identification of phages.

## Materials and Methods

### *C. difficile* isolates used in this study

The 91 *Clostridium difficile* 027 isolates used in this study were collated by Prof. Mark Wilcox, University of Leeds, UK. They were isolated from *C. difficile* toxin positive faecal samples that were submitted as part of routine diarrhoea surveillance in 9 hospitals in England. The isolates have previously been divided into 23 MLVA and 5 pulsovar types using MLVA and PFGE, respectively [6].

Other *C. difficile* ribotypes used in this study were 001, 002, 005, 014, 015 and 020. These were isolated from *C. difficile* toxin A and B positive faecal samples collected from University Hospitals of Leicester NHS Trust, Leicester, England. Faecal samples were added to a 50% solution of industrial methylated spirit in water, mixed thoroughly and left to stand at room temperature for 1 h. This material was then used to inoculate Braziers CCEY agar. *C. difficile* colonies were identified by their morphology, horse manure smell, yellow/green fluorescence under long wave uv light and agglutination using a latex agglutination test (Oxoid, Hampshire, UK).

### PCR ribotyping

Identified *C. difficile* colonies were subcultured on Brain Heart Infusion (BHI) (Oxoid, Hampshire, UK) medium supplemented with 7% defibrinated horse blood for 48 h. Bacterial DNA was extracted using Chelex 100 Molecular Biology Grade Resin (BioRad Laboratories, Carlifornia, USA) as previously described [6], [22]. PCR ribotyping was performed as described by Stubbs *et al.* with modifications [23]. *C. difficile* 16S–23S intergenic spacer regions were amplified using cdiffFP3 5′-CTGGGGTGAAGTCGTAACAAGG-3′ (forward) and cdiffRP4 5′-GCGCCCTTTGTAGCTTGACC-3′ (reverse) oligonucleotide primers. The reaction mixture was reduced in volume to ∼20 µl on a heating block at 75°C. PCR products were separated in 3% (w/v) RESponse Regular PCR Agarose gel (Bioplastics, The Netherlands) prepared in 1× Tris-acetate-EDTA (TAE, pH 8), containing 0.1 µl/ml GelRed (Biotium, Hayward, Carlifornia, USA). A 100-bp molecular standard (Fermentas, York, United Kingdom) was used and the gel was run in TAE buffer for 4 h (150 mA). *C. difficile* ribotypes were determined by visualizing on agarose gels and comparing resultant PCR ribotype profiles with those of known ribotypes (obtained from *C. difficile* Ribotyping Network for England and Northern Ireland reference laboratory at Leeds).

### Prophage induction

About 10 ml overnight BHI broth cultures of the 91 *C. difficile* 027 isolates were exposed to mitomycin C (Fisher Scientific, Loughborough, UK) or norfloxacin (Sigma-Aldrich, Dorset, UK) at a final concentration of 0.3, 1, 3, 6 and 9 µg/ml with shaking at 100 rpm for 24 h. Cultures were then centrifuged at 3400× g for 10 min. The resultant supernatants were passed through a 0.22 µm filters and stored at 4°C until further analysis.

### Transmission electron microscopy (TEM)

A 5 µl volume of each induced prophage suspension was placed on individual glow discharged pioloform/carbon coated copper grids which were allowed to stand for 5 min for bacteriophage to bind. The grids were blotted with Whatman filter paper, rinsed with 10 µl of double distilled water, blotted and stained with 10 µl of 1% w/v uranyl acetate. The stain was added one drop at a time, and allowed to stand for 5–10 s. Excess stain was removed by blotting leaving a thin film of suspension on the surface of the grids. The grids were allowed to dry for approximately 5 min and examined using JEOL 1220 electron microscope with an accelerating voltage of 80 kV. Digital images were captured using SIS Megaview III Digital camera with associated analysis software.

### Phage purification

*C. difficile* 027 broth cultures were induced and centrifuged in a 45 ml volume as previously described for prophage induction. The supernatants were decanted into sterile 50 ml centrifuge tubes and NaCl was added to a concentration of 1 M. The cultures were mixed and incubated on ice for 1 h. A 10% (w/v) of polyethylene glycol (PEG) 8000 (Fisher Scientific, New Jersey, USA) was slowly added with continuous stirring at 4°C until completely dissolved. The treated supernatants were stored at 4°C overnight and then centrifuged at 14, 334× g for 10 min at 4°C. The resultant pellets were resuspended in 1 ml of SM buffer (10 mM NaCl, 8 mM MgSO_4_.7H_2_O, 50 mM Tris-Cl, pH 7.5), washed with an equal volume of chloroform and centrifuged at 3, 398× g for 15 min. The upper aqueous phases were collected and filtered through 0.22 µm filters. The filtrates were used for PFGE analysis and DNA extraction.

### Pulsed-field gel electrophoresis (PFGE)

Agarose plugs were made by mixing 40 µl of PEG purified phage lysates and 60 µl 2% (w/v) Seaplaque CTG agarose (Cambrex Bio Science Wokingham, Berkshire, UK) in 0.5× Tris-Borate-EDTA (TBE, pH 8). This was dispensed into 120 µl capacity PFGE molds and solidified at 4°C for 30 min. The plugs were then removed and incubated in lysis buffer containing 100 mM EDTA, 100 mM Tris-Cl pH 9.0, 1% SDS, and 0.5 mg/ml proteinase K at 55°C overnight. After three washes in 1× TE (10 mM Tris-Cl pH 7.5, 1 mM EDTA pH 8.0), the plugs were placed in a 1% (w/v) Pulsed-field certified megabase agarose (BioRad Laboratories, Carlifornia, USA). The products were separated in 0.5× TBE buffer using a Bio-Rad CHEF-DR-II Pulsed-Field Electrophoresis System (Bio-Rad, Richmond, CA) at 6 V/cm for 15 h with a pulse ramp from 5–13 s at 14°C. The low range PFG marker (New England Biolabs, Herts, UK) was used as a molecular ladder. Gels were stained with GelRed (Biotium, Hayward, Carlifornia, USA). A digital image of the gel was captured using a SynGene camera assembly and GeneSnap software.

### Phage DNA extraction, primer design, PCR, cloning and sequencing

Phenol chloroform extraction and isopropanol precipitation was used for phage and genomic DNA extraction [24]. Four *C. difficile* phage sequences were used to design degenerate primers for the myovirus major capsid gene (Figure S1). Three of the four *C. difficile* gene sequences CD630, phiC2 and phiCD119 were obtained from NCBI and the fourth (phi12) was a partial DNA sequence of a *C. difficile* temperate myovirus kindly donated by Katherine Hargreaves (University of Leicester). A BLAST search for each of these sequences was performed to determine the specificity of the gene. The sequences were aligned using ClustalW and candidate sequences for the forward and reverse primers were manually selected between 11–29 nucleotide and 345–365 nucleotide regions respectively (Figure S1). The forward and reverse primers selected for targeting the capsid-encoding sequences were CDMCapF1 5′- CACTARMKTAYGSAMAAGWW-3′ and CDMCapR1 5′- CWRTAAGCATCYATCTCTGG -3′ respectively. Polymerase chain reactions were performed in a total volume of 50 µl, containing 0.25 mM dNTPs, 3 mM MgCl_2_, 2 µM forward and reverse primers, 1× PCR buffer, ∼50 ng of template DNA and 0.5 U of Taq polymerase (Bioline, London, UK). Amplification conditions were: 94°C for 2 min, 30 cycles of 94°C for 45 sec, 48°C for 45 sec, 72°C for 1 min, with a final extension of 10 min at 72°C. PCR products were gel-purified using NucleoSpin Extract II (Fisher Scientific, Leicestershire, UK), and subjected to TOPO TA cloning (Invitrogen, Paisley, UK). Plasmid extraction was performed using the Sigma GeneElute plasmid miniprep kit (Sigma-Aldrich, Dorset, UK). Plasmids were digested with EcoR1 (New England Biolabs, Herts, UK) to confirm the inserts and sequencing was carried out by GATC Biotech using M13 primers. Sequencing results were edited using Chromas 2.33 and translated into amino acids using Transeq sequence analysis in EMBL-EBI. The nucleotide and protein sequences were analysed using nucleotide and protein BLASTs respectively (NCBI) and phylogenetic analysis was carried out using MEGA 5.

## Results

### Morphological diversity of temperate *C. difficile* 027 bacteriophages

**Figure 1:**
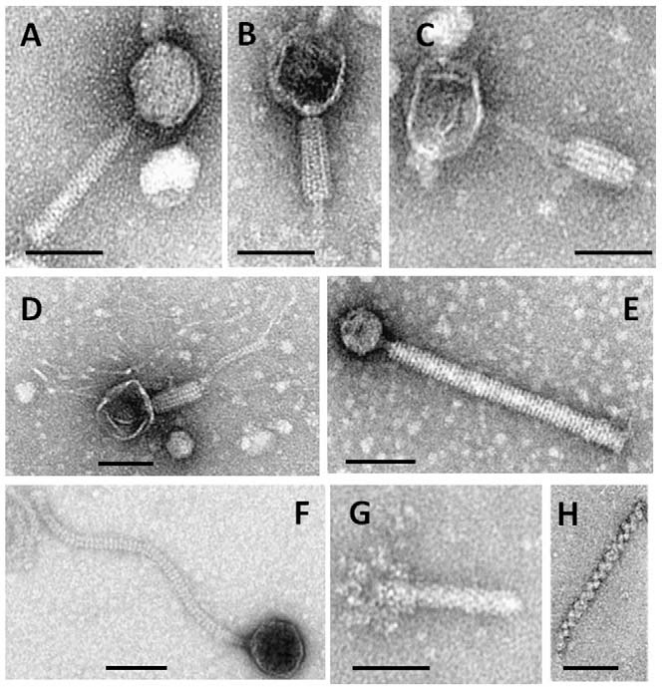
Morphological diversity of temperate bacteriophages associated with 91 *C. difficile* 027 isolates used in this study. Isolates were induced using mitomycin C or norfloxacin at a final concentration of 3 µg/ml. Prophages from the induced filtered lysates were analysed using TEM. Bars ∼70 nm. Measurement was estimated by measuring 6 phages in each sample.

TEM analysis following induction revealed a diverse set of phage morphologies associated with *C. difficile* ribotype 027, with a clear bias towards myoviruses, which were induced from 63 isolates (Figure 1, Table S1). Intact myoviruses (A) were only induced from two isolates and all had hexagonal capsids of ∼70 nm in diameter which were joined to the tail by a short portal protein (Figure 1). The tails were ∼200 nm by 20 nm with horizontal striations on the sheaths which end in 5–6 ∼50 nm long tail fibres. The two isolates yielding these intact myoviruses also yielded defective myoviruses (Morphologies B and C). These ‘defective myoviruses’ were the only viral particle morphology induced from a further 59 isolates (Table S1). These typically had contracted tail sheaths with the wider part of the sheath at various positions along the remaining tail tube (Morphologies B and C) (Figure 1). Some of the defective myoviruses had very long tail fibres such as those of morphology D (Figure 1). The remaining one isolate that yielded a defective myovirus also yielded a siphovirus. A novel *C. difficile* myovirus (E) was isolated from one isolate. This particle had a small hexagonal capsid of ∼40 nm diameter and tail of approximately 20 nm in diameter and 220 nm long (Figure 1). The capsid was joined to its tail by a thin portal protein like structure of ∼10 nm in length and width. The tubular tail had an average of 55 striations ±3 (based on an average of six) and the sheath ended with around 6–7 tail fibres.

Intact siphoviruses typified by F were released from three of the isolates (Figure 1, Table S1). These all had a hexagonal capsid ∼70 nm in diameter with a flexible ∼230–350 nm tail. Dual phage carriage was observed in four isolates; one isolate had a defective myovirus and a siphovirus (66L), two had a defective myovirus and an intact myovirus (96L and 91L) and finally one had two morphotypes of siphoviruses 48LM (Table S1). The two morphotypes of siphoviruses had the same capsid diameters but differed in their tail length. One had a tail length of ∼230 nm and the other a tail length of ∼350 nm (Table S1).

Twenty-five isolates produced phage tail-like particles only. There were two discrete categories of phage tail-like particles identified. One category (G) had particles which looked like genuine phage tails and were ∼160 nm by 20 nm terminating in horizontal protrusions (Figure 1). These phage tail-like particles were produced by all isolates (in addition to other morphologies) (Table S1). Two isolates harboured particles with morphology belonging to the second category (H) (Figure 1). This particle type had tightly coiled spirals approximately 20 nm diameter and between 80–350 nm long. Only one isolate contained no prophage particles.

### Norfloxacin and mitomycin C prophage induction

To optimize prophage induction, five concentrations (0.3, 1, 3, 6 and 9 µg/ml) of norfloxacin and mitomycin C were used on ten of our samples. All of the induced cultures released prophages with 0.3, 1 and 3 µg/ml of antibiotic and there was an increase in phage abundance within this antibiotic concentration range. Although most cultures were poorly induced at 6 µg/ml, one sample (16L) produced the highest phage abundance at this concentration. All cultures were killed after 24 h of induction at 9 µg/ml and no phages were observed in these culture filtrates. We therefore established that 3 µg/ml concentration of either antibiotic is optimal for *C. difficile* phage induction. We also observed that morphologies of the induced phage particles did not vary with antibiotic concentration.

The growth responses of the 91 induced cultures and the efficacy of the two antibiotics (at 3 µg/ml) to cause prophage induction were compared (Figure S2, Table S1). Intact phages were induced from approximately 61% of the isolates with both antibiotics. However, 8% and 31% of the isolates yielded intact phages with mitomycin C and norfloxacin, respectively. In four isolates, mitomycin C and norfloxacin induced different prophages from their hosts. Isolate 48L yielded two siphoviruses morphotypes following mitomycin C induction, but only one morphotype following norfloxacin induction. Similarly, isolate 66L yielded two different phage morphologies (a siphovirus and a defective myovirus) with norfloxacin but only a defective myovirus with mitomycin C. Isolate 96L yielded a myovirus and a defective myovirus when induced with norfloxacin and phage tail-like particles with mitomycin C. Finally, isolates 91L yielded a myovirus with mitomycin C but induction with norfloxacin yielded a myovirus and a defective myovirus (Table S1).

### Correlation of phage carriage with ribotype 027 subtypes

A correlation between phage carriage and the MLVA types was observed in this study (Table 1). MLVA 1–15 yielded defective myoviruses apart from three isolates (96L, 66L and 91L) which in addition to defective myoviruses contained a myovirus (96L, 91L) or a siphovirus (66L). MLVA 16, 17, 19, 20, 21, 22 and 23 were found only to contain phage tail-like particles with one exception each in MLVA 16 (isolate 16L contained a novel myovirus E) and MLVA 22 (isolate 36L which had no phage particles under the induction conditions above). There were three isolates classified as MLVA 18 and two had inducible siphoviruses and one, a defective myovirus (Table 1, Table S1). Pulsovar I (containing 14 different MLVA types, 53 isolates) was strongly associated with the production of defective myoviruses. All contained defective myoviruses except isolate 71L which contained only phage tail-like particles. Pulsovar II was only represented by one isolate and contained a defective myovirus. Two isolates from Pulsovar III were examined and both contained siphoviruses (Table 2, Table S1). Thirty-three isolates from Pulsovar IV were examined and they all yielded phage tail-like particles. In addition, ten isolates also yielded defective myoviruses. Pulsovar V consists of two isolates; one contained phage tail-like particles and the other (36L) contained no phage.

**Table 1:** Phage carriage in 91 *C. difficile* 027 isolates in relation to their MLVA types.

MLVA type	Number of isolates	Morphology of phage isolated	Exceptions
1	3	Defective myovirus	-
2	1	Defective myovirus	-
3	5	Defective myovirus	-
4	7	Defective myovirus	-
5	1	Defective myovirus	-
6	2	Defective myovirus	-
7	7	Defective myovirus	-
8	2	Defective myovirus	-
9	2	Defective myovirus	-
10	1	Defective myovirus	-
11	3	Defective myovirus	-
12	6	Defective myovirus	66L (Siphovirus F)
13	12	Defective myovirus	96L (Myovirus A)
14	3	Defective myovirus	-
15	6	Defective myovirus	91L (Myovirus A)
16	15	PT- LPs*	16L (Novel myovirus E)
17	5	PT- LPs*	-
18	3	Siphovirus	53L (Defective myovirus B)
19	1	PT- LPs*	-
20	2	PT- LPs*	-
21	1	PT- LPs*	-
22	2	PT- LPs*	36L (No phage)
23	1	PT-LPs*	-

* , Phage tail-like particles.

Prophage carriage among the 91 *C. difficile* 027 isolates induced with mitomycin C or norfloxacin was correlated to their multiple-locus variable number tandem repeat analysis (MLVA) types. MLVA 1–15 yielded defective myoviruses with three exceptions in MLVA 12 (isolate 66L yielding a siphovirus F), MLVA 13 (isolate 96L yielding a myovirus A) and MLVA 15 (isolate 91L yielding a myovirus A) in addition to the defective myoviruses. MLVA 16 and 17 and 19–23 all yielded phage tail-like particles (PT-LPs) except in MLVA 16 with one isolate yielding myovirus E and another (isolate 36L) in MLVA 22 which yielded no phage under the experimental conditions. Among the three isolates examined in MLVA 18, two yielded siphoviruses and one (isolate 53L) yielded defective myoviruses.

**Table 2:** Phage carriage in 91 *C. difficile* 027 isolates in relation to their different pulsovar types.

Pulsovar type	Number of isolates	Morphology of phage isolated	Exceptions
I	53	Defective myovirus	71L (PT-LPs)*
II	1	Defective myovirus	-
III	2	Siphovirus	-
IV	33	PT-LPs*	10 isolates (Myovirus A, E and defective myovirus B, C)
V	2	PT-LPs*	36L (No phage)

* , Phage tail-like particles.

Prophage carriage among the 91 *C. difficile* 027 induced using mitomycin C or norfloxacin was also correlated to their pulsovar types. Pulsovar types I and II yielded defective myoviruses with one exception in Pulsovar I (isolate 71L) which yielded only phage tail-like particles (PT-LPs). Pulsovar type III yielded siphoviruses. Pulsovar types IV and V yielded phage tail-like particles with ten exceptions in Pulsovar type IV and one in Pulsovar type V.

### Genetic diversity of *C. difficile* 027 temperate bacteriophages

**Figure 2:**
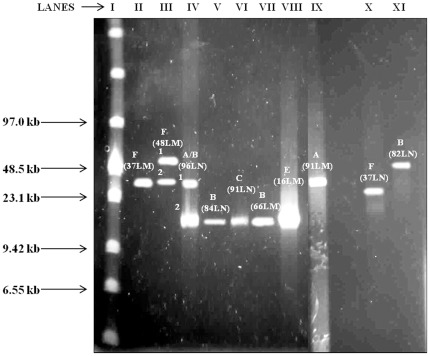
Pulsed-field gel electrophoresis analysis of whole temperate phage genomes showed diverse sizes. Pulsed-field gel electrophoresis was used to analyse the PEG purified phage lysates. All the defective myoviruses with morphology B and C in samples 96LN2, 84LN, 91LN, 66LM and novel myovirus E (16LM) had genome size of ∼15 kb. The siphovirus F in 48LM2 and 37LM and typical myovirus A in samples 96LN1 and 91LM had genome size of ∼30 kb. Only the siphovirus in 48LM1 and defective myovirus in 82LN had genome size of ∼50 kb. Samples with suffix N, M indicates isolates induced with norfloxacin or mitomycin C respectively.

PFGE analysis revealed that phage genome size ranged from ∼15 to 50 kb (Figure 2). The smallest genomes were seen in the defective myoviruses B and C from isolates 96LN2, 84LN, 91LN, and 66LM (Lanes IV, V, VI and VII respectively) and the novel myovirus E (lane VIII). (Suffixes N and M symbolise isolates induced with norfloxacin and mitomycin C, respectively). Typical myoviruses A induced from isolates 96LN1 and 91LM (Lanes IV and IX respectively) had a genome size of ∼30 kb as well as the siphoviruses isolated from 37LM (Lane II), 48LM2 (Lane III) and 37LN (Lane X). Only the siphovirus isolated from 48LM1 and one defective myovirus isolated from 82LN had a large genome size of ∼50 kb (Lanes III and XI respectively). Two isolates (48LM and 96LN) shown to previously have dual phage carriage exhibited genomes of two distinct sizes, 50 and 30 kb for 48LM1 and 48LM2 (Lane III) and 30 and 15 kb for 96LN1 and 96LN2 (lane IV) respectively. This provides good evidence of their dual phage status. On the other hand, morphologically distinct phages that were induced from the same isolate but using different antibiotics showed different genome sizes (91LM with ∼30 kb genome size and 91LN ∼15 kb). Furthermore, in all cases examined, the siphoviruses which were morphologically identical under TEM also showed identical genome sizes (for example 48LM2, 37LN and 37LM having genome size of ∼30 kb).

### Phylogenetic analysis of *C. difficile* phages

**Figure 3:**
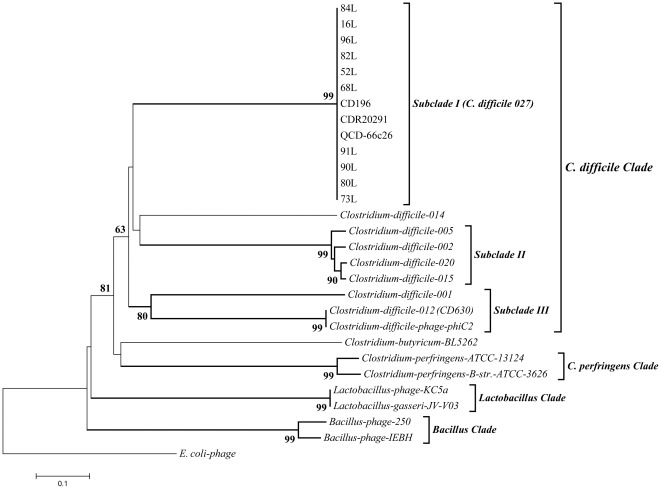
Evolutionary relationship of *Clostridium difficile* based on the myovirus capsid gene. The evolutionary history was inferred using the Neighbor-Joining method. The percentage of replicate trees in which the associated taxa clustered together in the bootstrap test (1000 replicates) is shown next to the branches. The evolutionary distances were computed using the p-distance method based on their amino acid sequences. All positions containing gaps and missing data were eliminated. The analysis involved 29 amino acid sequences of 10 (84L, 16L, 96L, 82L, 52L, 68L, 91L, 90L, 80L and 73L) representative isolates of the ribotype 027 subclades and seven other ribotypes (ribotypes 014, 005, 002, 020, 015 and 001). Five other sequences including CD196, CDR20291 and QCD-66c26 (ribotype 027), CD630 (ribotype 012) and phiC2 were obtained from *in-silico* PCR. Other sequences were obtained from NCBI searches. All sequences with 75% similarities were assigned into a subclade. Evolutionary analyses were conducted in MEGA5.

PCR was performed on a range of *C. difficile* clinical isolates including ten *C. difficile* 027 and six other ribotypes (014, 005, 002, 020, 015 and 001) to amplify the capsid gene of the phage, followed by cloning and sequencing of the inserts. In all the isolates examined the portion of the capsid gene sequence was 358 nucleotides which covers 40% of the gene. The degenerate primers were also used to amplify genomes of three other *C. difficile* 027 strains (CD196, QCD-66c26 and R20291), ribotype 012 (CD630) and phiC2 using *in-silico* PCR (http://insilico.ehu.es/PCR/). Results indicated that the primers were specific to *C. difficile* myoviruses or lysogenic hosts harbouring these prophages. Nucleotide BLAST (blastn) combined with protein BLAST (blastp) confirmed the capsid sequences. Phylogenetic analysis of both the nucleotide and amino acid sequences showed that all *C. difficile* isolates cluster together, forming the *C. difficile* clade. Within this, all *C. difficile* 027 strains (including CD196, QCD-66c26 and R20291 from *in-silico* PCR) have identical sequences and therefore form a single subclade (Subclade I) (Figure 3). Two other distinct subclades can be defined from the analysis of the capsid gene, subclade II and III. Subclade II is comprised of ribotypes 005, 002, 020 and 015 and Subclade III is comprised of *C. difficile* 001, *C. difficile* 012 (CD630) and *C. difficile* phage phiC2 [17], [21]. Within Subclade III *C. difficile* 012 (CD630) and phiC2 formed a much tighter clade [17]. The ribotype 014 sequence falls outside the other *C. difficile* ribotypes. Regarding the outgroup taxa; the *E.coli* phage, *C. perfringens*, *Lactobacillus* and *Bacillus* clades are distinct from each other and from the *C. difficile* clade (Figure 3).

## Discussion

Given the excess morbidity and mortality associated with *C. difficile* ribotype 027, we investigated prophage content as a factor that may be contributing to its diversity and virulence. Bacteriophage carriage within a single *C. difficile* ribotype has not been extensively examined. Previous work examined six isolates from different *C. difficile* ribotypes and found that each ribotype harboured a morphologically and genetically different prophage and suggested a correlation between the type of prophage and ribotype [14]. However, the examination of 91 ribotype 027 isolates show that prophage carriage within *C. difficile* 027 is morphologically and genetically variable and therefore may contribute to diversity.

The morphology of the putative myovirus A identified in our study is similar to the temperate phage induced from a clinical isolates of *C. difficile* 027 and to phages found in unknown ribotypes [13], [25]. To our knowledge, this is the only temperate bacteriophage morphology previously reported to be associated with ribotype 027. However, despite the capsid and tail diameters being similar to the previously described phage, the viruses described here have longer tail lengths suggesting that they may be different [12], [14]. The defective myoviruses B, C and D isolated from the majority of our lysates have also previously been shown to be associated with mitomycin C induced lysates which also contained typical myoviruses [14]. However the majority of lysates examined here only had these defective myoviruses. Although it is assumed that phage tail sheaths contract when infecting a host and ejecting DNA, our TEM analysis did not confirm this. Our data therefore support previous observations that *C. difficile* carries two morphologies of myoviruses [12].

The novel *C. difficile* myovirus E identified from one isolate is similar to ‘killer particles’ found in *Bacillus* species, *Acetobacter* species and *Clostridium botulinum* [26], [27]. These particles are thought to package host DNA instead of bacteriophage DNA and are able to kill sensitive hosts but not replicate within them [26], [28]. We have made several attempts to propagate this phage through plaque assays and spot tests but unfortunately, no host was found despite testing them on 63 different *C. difficile* hosts (including clinical and environmental isolates). However, myovirus E was able to clear lawn of one host (CD630) but scrapped zones of killing were not able to further propagate, and TEM observation on these lysates could not identify whole phages (Figure S3). Therefore work is on-going to further characterise this phage and its possible impact on the pathogenicity of its host.

Other temperate bacteriophage morphology identified here are the siphoviruses typified by F. Although, siphoviruses with dimensions similar to those isolated here have previously been reported on other ribotypes of *C. difficile*, this report is the first to show the association of this phage morphology to ribotype 027 [9], [13], [14], [25]. Interestingly, two of the three siphoviruses we isolated were induced from isolates within a pulsovar subtype associated with very high severity of CDI (death within the first thirty days of infection) [6]. This suggests their possible role in the pathogenicity of 027 strains. Furthermore, a previous report has shown that infection of *C. difficile* ribotype 027 by phiCD38-2 (a temperate *C. difficile* siphovirus) resulted in the increase expression of toxin A and B [9]. Although bioinformatics analysis of phiCD38-2 has failed to identify toxins or virulence factors in the viral genome, the phage has been shown to interact with the *C. difficile* pathogenicity locus (PaLoc) thus having the potential to influence toxin production. Therefore, phage detection may represent a useful marker to identify strains that are associated with increased disease severity, and this early detection could aid clinical practice.

Dual phage carriage has previously been reported in CD630, CD8 and CD38 in which two morphologically and genetically distinct phages were induced from a single *C. difficile* strain [14], [17]. However, CD630, CD8 and CD38 strains were of different ribotypes. Therefore, this study is the first to show two inducible and morphologically different phages with different genome sizes from ribotype 027 isolates. The correlation of phage carriage with MLVA types of ribotype 027 suggests that it is stable within this ribotype and does contribute to its diversity and severity of CDI [29].

The presence of phage tail-like particles, such as those represented by G in all our induced lysates, has previously been reported to be found in several induced lysates of *C. difficile* [14], [25]. However, the lengths of the observed phage tail-like particles in this study were longer than those in previous reports (an average size of 160 nm as opposed to 130 nm). These phage tail-like particles have morphology similar to bacteriocin of *Budvicia aquatica* and *Pragia fontium* and phage tail-like particles of *Vibrio spp* [30], [31]. Another phage-like particles typified by H in two mitomycin C induced samples, have a morphology that has not previously been observed to be associated with *C. difficile* but they do closely resemble a bacteriocin induced from *Pseudomonas aeruginosa* p28 [32], [33], [34]. Some of the bacteriocins have been shown to have bactericidal activities and provide competitive advantage to their hosts [34], [35]. Although the phage tail-like particles G have been isolated from *C. difficile* isolates, their biological functions have not yet been determined. However, we found them present in all our induced isolates (in addition to other phage morphologies). This suggests their potential role in the survival of these isolates as earlier suggested [34], [35]. Since their tails are non-contractile and their capsid DNA sequences show significant homology with myovirus sequences during *in-silico* PCR, our report concurs with the previous study that these particles may share some evolutionary characteristics with the myoviruses [36].

It is evident from the results of this study that the temperate phages isolated also have diverse genome sizes. The DNA size of ∼15 kb (96LN2, 84LN, 91LN and 66LM) observed here are amongst the smaller sizes for phages isolated from *Clostridium* species [37]. The genome sizes of temperate bacteriophages from isolates 91LM, 96LN1 (myoviruses) and 82LN (defective myovirus) are in agreement with the genome sizes of temperate bacteriophages of *C. difficile* belonging to *Myoviridae*, having genomes varying in size from 29–160 kb [17], [18], [37]. The genome sizes of the siphoviruses isolated from 37L and 48L are in agreement with previous reports [9], [20]. We tested ten restriction endonucleases BamH1, Nde1, BstB1, HindIII, EcoR1, Sty1, Sma1, Sau3A1, Mbo1 and Dpn1 on the DNA of these phages. However, none of these restriction enzymes could digest the DNA from these phages, or if they digest the DNA, it was only partial so they did not result in suitable banding patterns to allow genomic comparisons. This is likely to be attributed to DNA methylases which are found in the genomes of *C. difficile* strains and which provide immunity to the restriction enzymes [18], [38].

The characterisation of phages by their morphology, genome size and inducibility is vital to understanding phage dynamics but these methods are labour intensive and dependent on induction antibiotic choice. Molecular markers to establish bacteriophage diversity and carriage would be of clear interest in terms of *C. difficile* host-phage dynamic characterisation. Such information would greatly enhance morphological studies.

Since prophages integrate into their host genomes, the use of these primers on genomic DNA will help to detect phages in isolates, thus direct strain choice before the time consuming phage induction procedures and TEM analysis. Phylogenetic analysis was done both at the nucleotide and the amino acid level and both trees gave the same supported tree topology within *C. difficile* phages. The amino acid sequences however allowed us to align the sequences from the *C. difficile* phages with phages which infect several other taxa and thus the analysis could be contextualised with respect to other phages. The ability of all the 027 isolates used in this study to form a strong grouping both at the nucleotide and amino acid level showed the much conserved nature of the genes of this ribotype [21]. This result is consistent with a previous report where DNA microarrays and Bayesian-based algorithms were used to phylogenetically analyse whole genomes of 75 *C. difficile* strains. They observed that the nineteen 027 strains studied appeared to form a tight group which was distinct from the other 56 strains [39]. This is in agreement with previous work in which it was found that *C. difficile* 027 strains have very conserved genes, are descended from a single ancestor and acquire diverse external genetic element such as prophages, plasmids, antibiotic resistant genes and mutations via horizontal gene transfer during evolution [21]. These factors are likely to contribute to their pathogenicity [38]. The *C. difficile* phage group which is distinct from other members of the Clostridial family and other bacterial species suggests that the capsid is a good molecular marker to indicate phage presence and study phylogenetic relationships in bacterial species.

Clearly, *C. difficile* 027 isolates contain morphologically and genetically diverse sets of bacteriophages which are inducible with specific antibiotics. The carriage of different bacteriophages by *C. difficile* 027 isolates shows that there is significant variation within the mobile genetic elements associated with this ribotype. The correlation of phage carriage with MLVA and pulsovar types within *C. difficile* 027 also suggests that these bacteriophages contribute to its diversity, and in turn contribute to the success of particular strains in terms of disease severity. Future work will aim to determine how bacteriophages contribute to the spectrum of disease severity observed with different *C. difficile* ribotypes.

## Supporting Information

Table S1 **Table showing diverse phage morphologies isolated from mitomycin C and norfloxacin inductions of 91** ***C. difficile*** **027 isolates belonging to different subclades.** Morphologies were identified using TEM. Bar ∼70 nm based on the measurement of six phages in each sample.
(DOC)

Figure S1 **Multiple alignment of four** ***C. difficile*** **phage sequences used in the design of the capsid primers.** DNA sequences of PhiC2, CD630 and phiCD119 were obtained from NCBI searches and phi12 was a partial DNA sequences provided by Katherine Hargreaves, University of Leicester. The sequences were aligned using ClustalW. Regions for the forward and reverse primers were manually selected and indicated using arrows.
(TIF)

Figure S2 **Graph showing different patterns of growth responses of** ***C. difficile*** **cultures during prophage inductions.** Overnight broth cultures of *C. difficile* 027 isolates in BHI at OD_550_ ∼1.2 were induced with norfloxacin or mitomycin C at final concentration of 3 µg/ml for 24 h. Final OD_550_ were taken at the end of 24 h. The effect of antibiotics on the growth of bacterial cultures was determined by measuring the OD_550_ values before and after induction. The growth responses all fitted into one of four patterns (A–D) and this was compared to phage release. I. For pattern A, there was a drop in OD_550_ with both mitomycin C and norfloxacin inductions. Although this pattern has previously been reported in *C. difficile* inductions, only 35 of the 91 isolates used in this study showed this profile. Furthermore, from these 35 isolates, 33 were found to harbour intact phages with the remaining two harbouring phage tail-like particles following induction by either antibiotic. II. For pattern B, the OD_550_ dropped with mitomycin C induction but increased following norfloxacin induction. Twenty-eight of isolates showed this pattern. The TEM analysis showed that there was phage release from 16 of the isolates following mitomycin C and 11 with norfloxacin induction. One isolate was found to contain no phages. III. Pattern C was the opposite of pattern B with OD_550_ increasing following mitomycin C induction but dropping following norfloxacin induction. Only five isolates showed this pattern. There was phage release from two isolates with mitomycin C and three with norfloxacin induction as confirmed by TEM analysis. IV. For pattern D, the OD_550_ remained relatively constant following either norfloxacin or mitomycin C induction of the cultures. This pattern was observed in 23 isolates. Only phage tail-like particles were observed with these isolates. In addition to the 24 h observations, 15 isolates belonging to the different patterns were selected and monitored after an induction time of 72 h. By this time point, all the 15 cultures showed a significant OD_550_ drop and a concomitant release of phages.
(TIF)

Figure S3 **Picture showing plaques of novel myovirus E on lawn of CD630.**
(TIF)
