# Supplementary material for: Diverse Temperate Bacteriophage Carriage in Clostridium difficile 027 Strains
Source: PLoS One. 2012 May 18;7(5):e37263. doi: 10.1371/journal.pone.0037263 (PMC3356267; doi:10.1371/journal.pone.0037263)
Supplement: Table S1 — Table showing diverse phage morphologies isolated from mitomycin C and norfloxacin inductions of 91 C. difficile 027 isolates belonging to different subclades. Morphologies were identified using TEM. Bar ∼70 nm based on the measurement of six phages in each sample. (DOC) [file pone.0037263.s001.doc]

| **Sample ID** | **MLVA** | **Pulsovars** | **Mitomycin C** | **Norfloxacin** |
| --- | --- | --- | --- | --- |
| 14L | 1 | I | 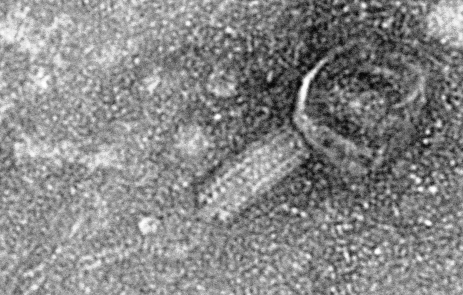 | 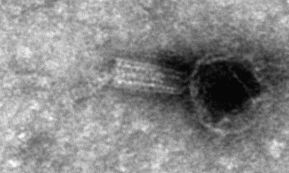 |
| 52L | 1 | I | 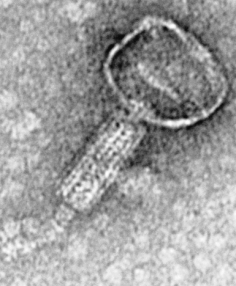 | 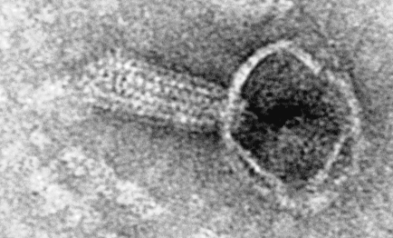 |
| 38L | 1 | I | 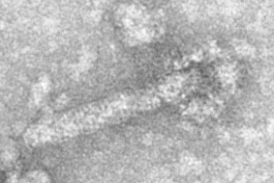 | 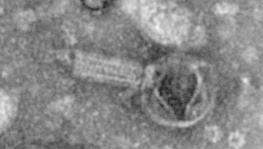 |
| 2L | 2 | I | 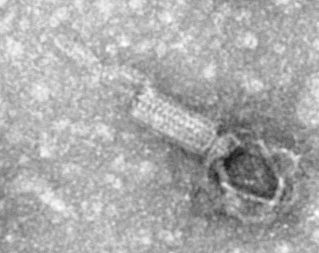 | 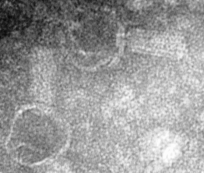 |
| 12L | 3 | I | 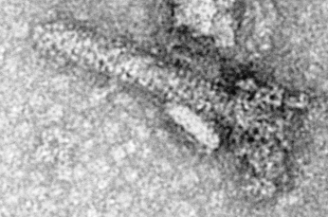 | 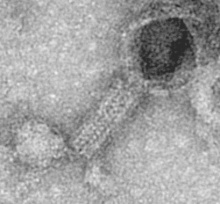 |
| 87L | 3 | I | 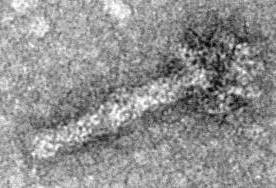 | 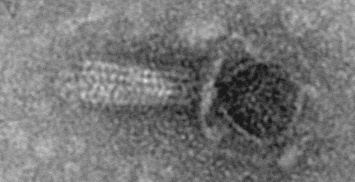 |
| 10L | 3 | I | 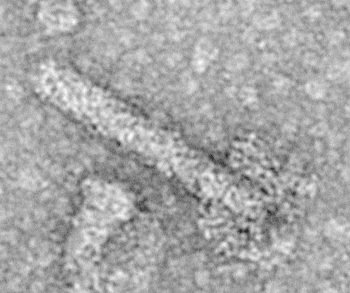 | 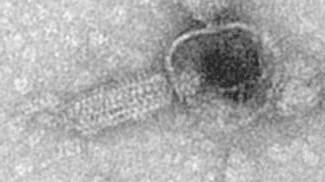 |
| 79L | 3 | I | 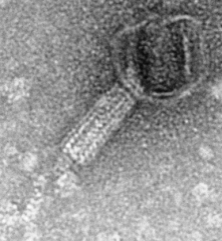 | 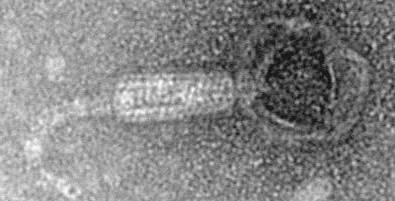 |
| 28L | 3 | I | 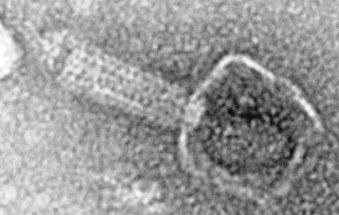 | 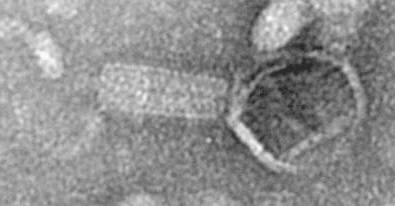 |
| 83L | 4 | I | 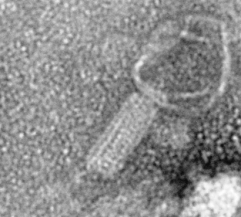 | 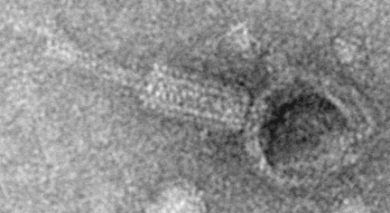 |
| 92L | 4 | I | 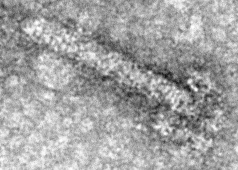 | 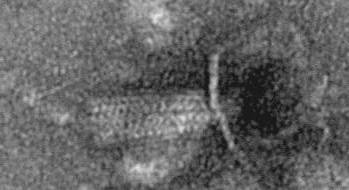 |
| 85L | 4 | I | 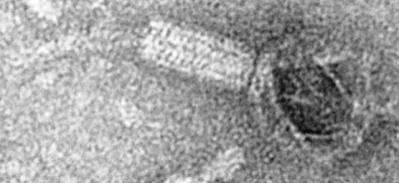 | 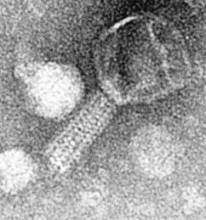 |
| 88L | 4 | I | 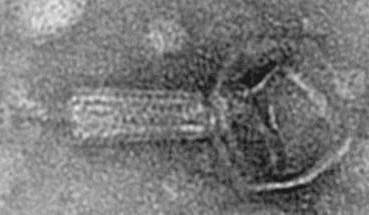 | 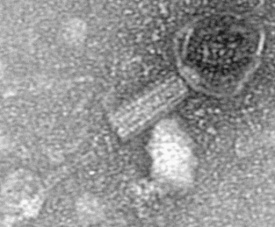 |
| 89L | 4 | I | 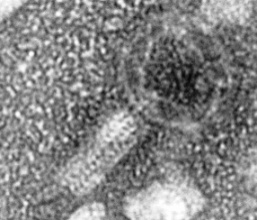 | 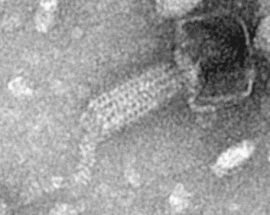 |
| 29L | 4 | I | 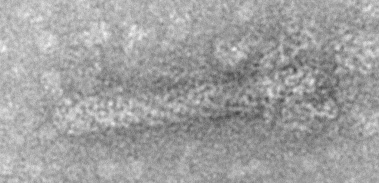 | 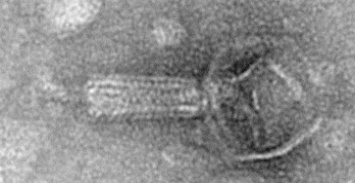 |
| 78L | 4 | I | 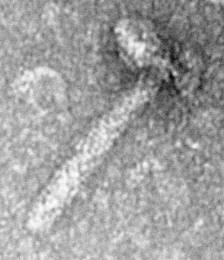 | 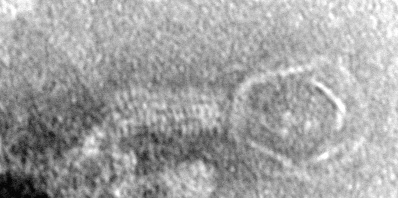 |
| 32L | 5 | I | 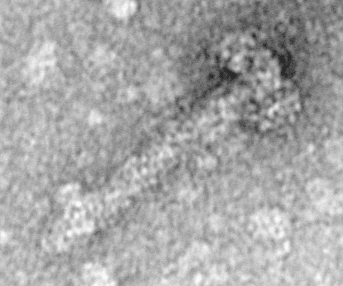 | 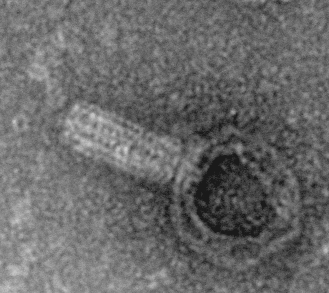 |
| 17L | 6 | I | 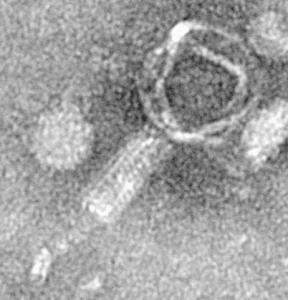 | 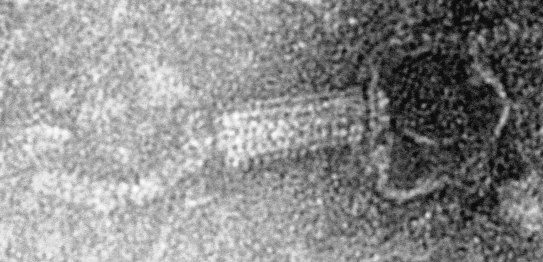 |
| 81L | 6 | I | 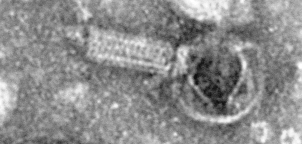 | 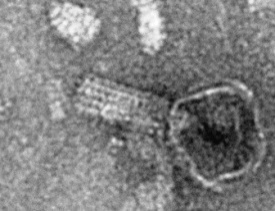 |
| 67L | 7 | I | 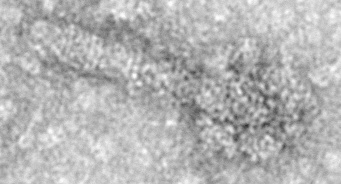 | 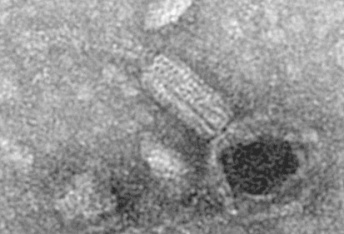 |
| 61L | 7 | I | 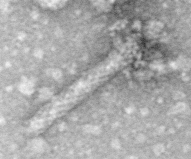 | 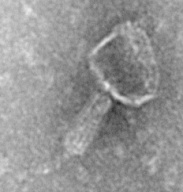 |
| 54L | 7 | I | 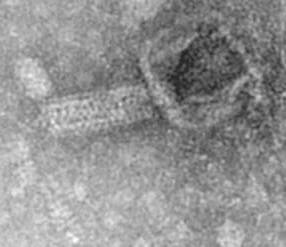 | 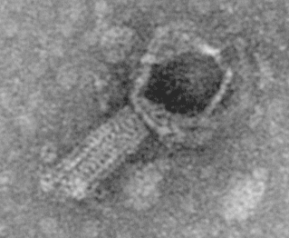 |
| 75L | 7 | I | 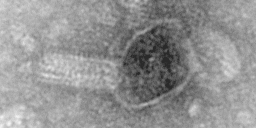 | 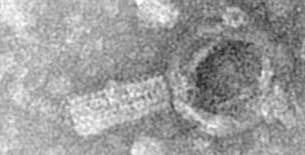 |
| 25L | 7 | I | 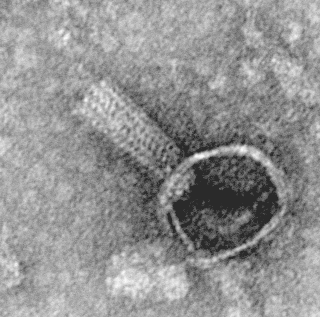 | 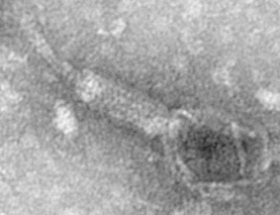 |
| 77L | 7 | I | 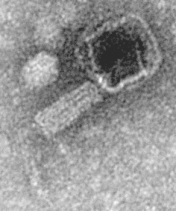 | 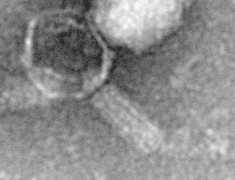 |
| 21L | 7 | I | 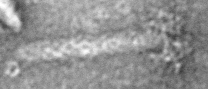 | 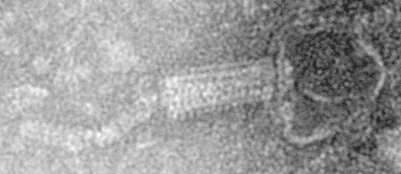 |
| 5L | 8 | I | 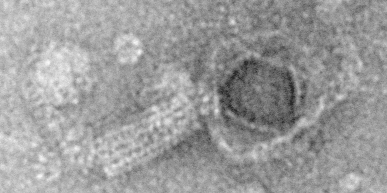 | 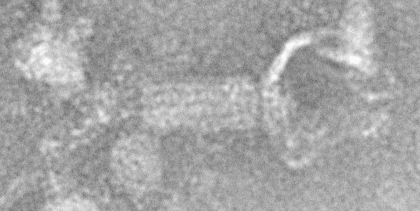 |
| 9L | 8 | I | 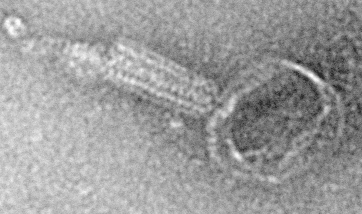 | 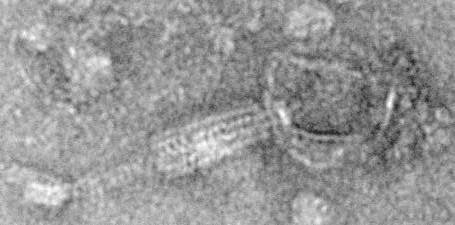 |
| 26L | 9 | I | 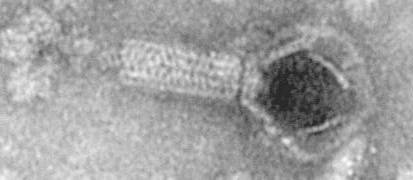 | 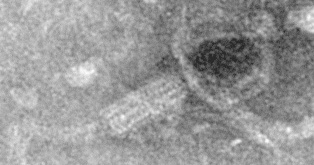 |
| 82L | 9 | I | 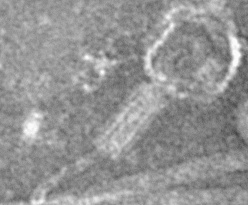 | 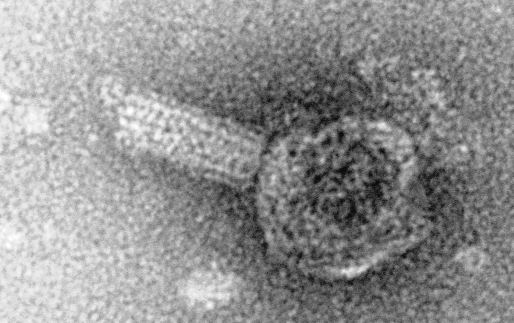 |
| 94L | 10 | I | 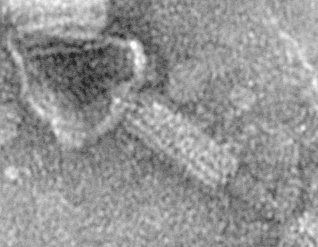 | 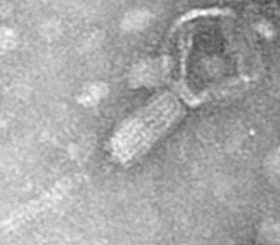 |
| 90L | 11 | I | 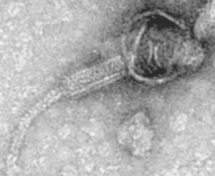 | 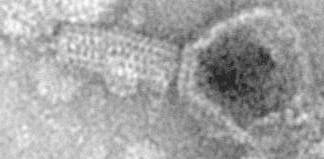 |
| 86L | 11 | I | 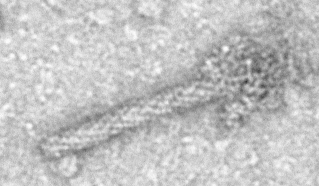 | 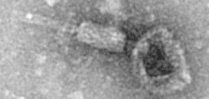 |
| 95L | 11 | I | 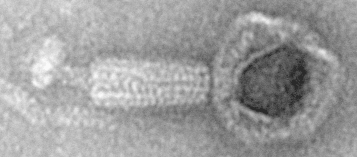 | 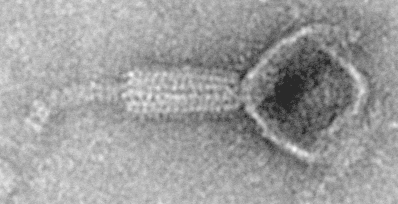 |
| 60L | 12 | I | 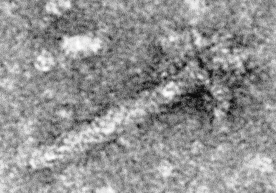 | 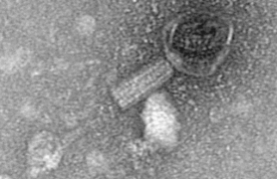 |
| 63L | 12 | I | 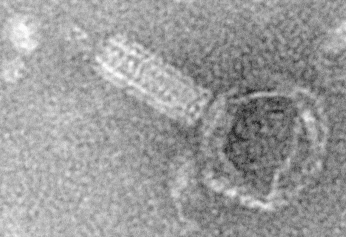 | 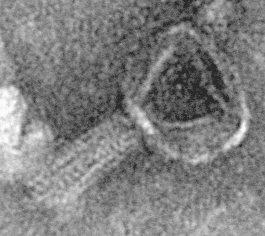 |
| 65L | 12 | I | 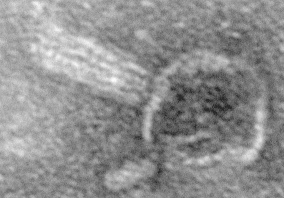 | 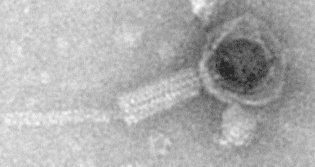 |
| 7L | 12 | I | 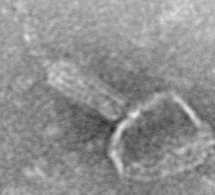 | 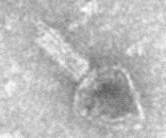 |
| 66L | 12 | I | 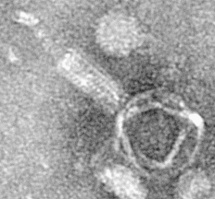 | 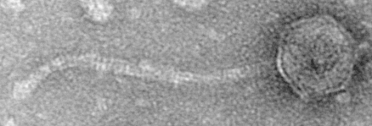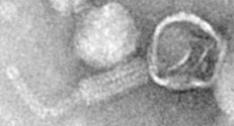 |
| 39L | 12 | I | 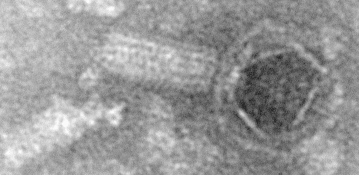 | 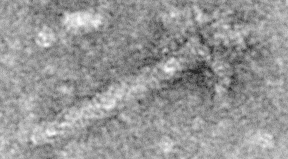 |
| 22L | 13 | I | 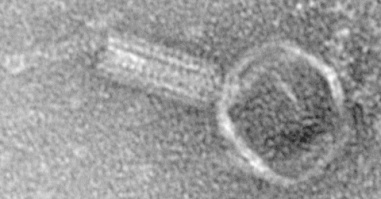 | 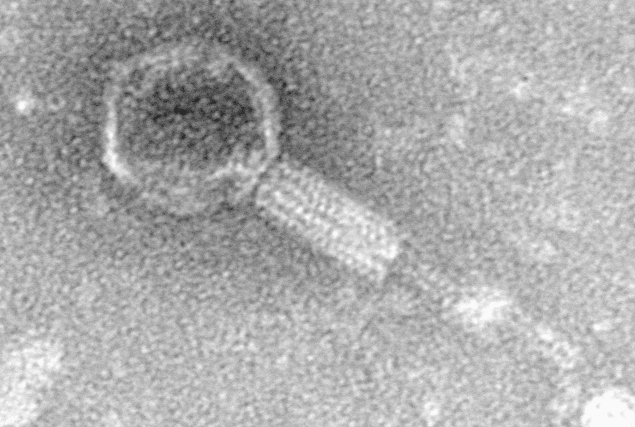 |
| 96L | 13 | I | 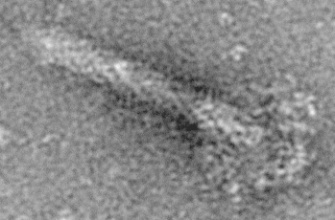 | 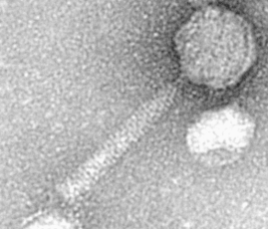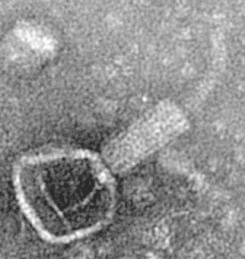 |
| 6L | 13 | I | 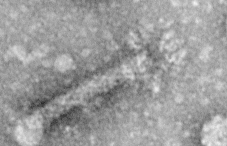 | 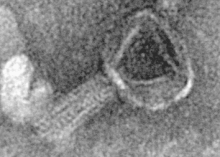 |
| 68L | 13 | I | 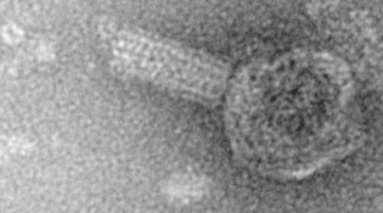 | 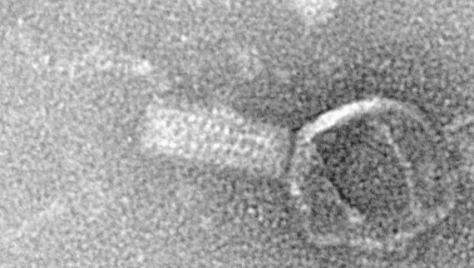 |
| 93L | 13 | I | 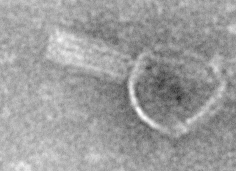 | 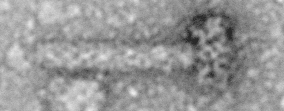 |
| 69L | 13 | I | 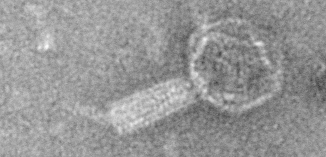 | 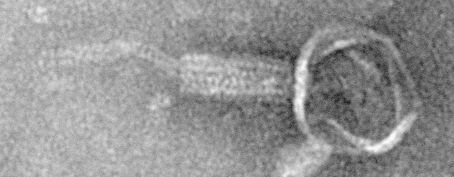 |
| 73L | 13 | I | 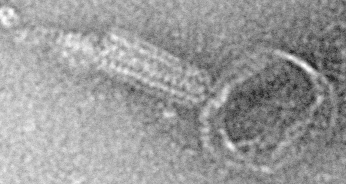 | 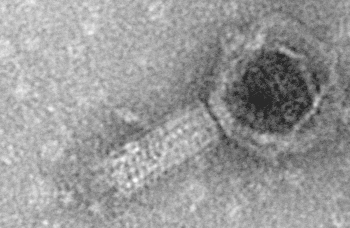 |
| 84L | 13 | I | 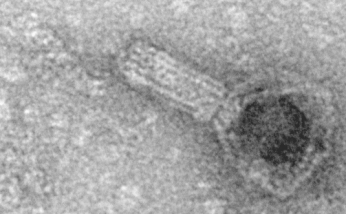 | 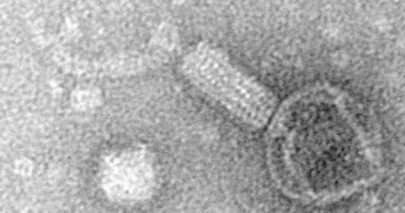 |
| 4L | 13 | I | 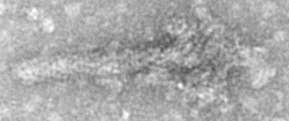 | 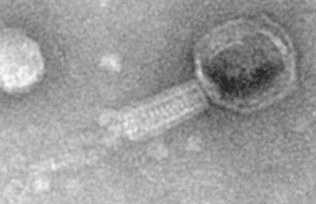 |
| 70L | 13 | I |  |  |
| 80L | 13 | I |  |  |
| 46L | 13 | I |  |  |
| 23L | 14 | IV |  |  |
| 47L | 14 | IV |  |  |
| 57L | 14 | IV |  |  |
| 31L | 15 | IV |  |  |
| 33L | 15 | IV |  |  |
| 34L | 15 | IV |  |  |
| 59L | 15 | IV |  |  |
| 20L | 15 | IV |  |  |
| 91L | 15 | IV |  |  |
| 3L | 16 | IV |  |  |
| 55L | 16 | IV |  |  |
| 1L | 16 | IV |  |  |
| 42L | 16 | IV |  |  |
| 15L | 16 | IV |  |  |
| 16L | 16 | IV |  |  |
| 19L | 16 | IV |  |  |
| 30L | 16 | IV |  |  |
| 35L | 16 | IV |  |  |
| 56L | 16 | IV |  |  |
| 50L | 16 | IV |  |  |
| 76L | 16 | IV |  |  |
| 18L | 16 | IV |  |  |
| 43L | 16 | IV |  |  |
| 44L | 16 | IV |  |  |
| 49L | 17 | IV |  |  |
| 71L | 17 | 1 |  |  |
| 58L | 17 | IV |  |  |
| 62L | 17 | IV |  |  |
| 64L | 17 | IV |  |  |
| 37L | 18 | III |  |  |
| 51L | 18 | II |  |  |
| 48L | 18 | III |  |  |
| 13L | 19 | IV |  |  |
| 53L | 20 | IV |  |  |
| 45L | 20 | IV |  |  |
| 40L | 21 | IV |  |  |
| 36L | 22 | V | No phage | No phage |
| 41L | 22 | V |  |  |
| 72L | 23 | IV |  |  |
