# Supplementary figures and images for: Diverse Temperate Bacteriophage Carriage in Clostridium difficile 027 Strains
Source: PLoS One. 2012 May 18;7(5):e37263. doi: 10.1371/journal.pone.0037263 (PMC3356267; doi:10.1371/journal.pone.0037263)

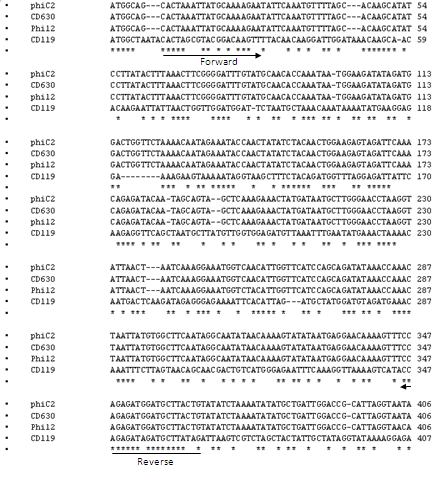

Supplement: Figure S1 — Multiple alignment of four C. difficile phage sequences used in the design of the capsid primers. DNA sequences of PhiC2, CD630 and phiCD119 were obtained from NCBI searches and phi12 was a partial DNA sequences provided by Katherine Hargreaves, University of Leicester. The sequences were aligned using ClustalW. Regions for the forward and reverse primers were manually selected and indicated using arrows. (TIF) [file pone.0037263.s002.tif]

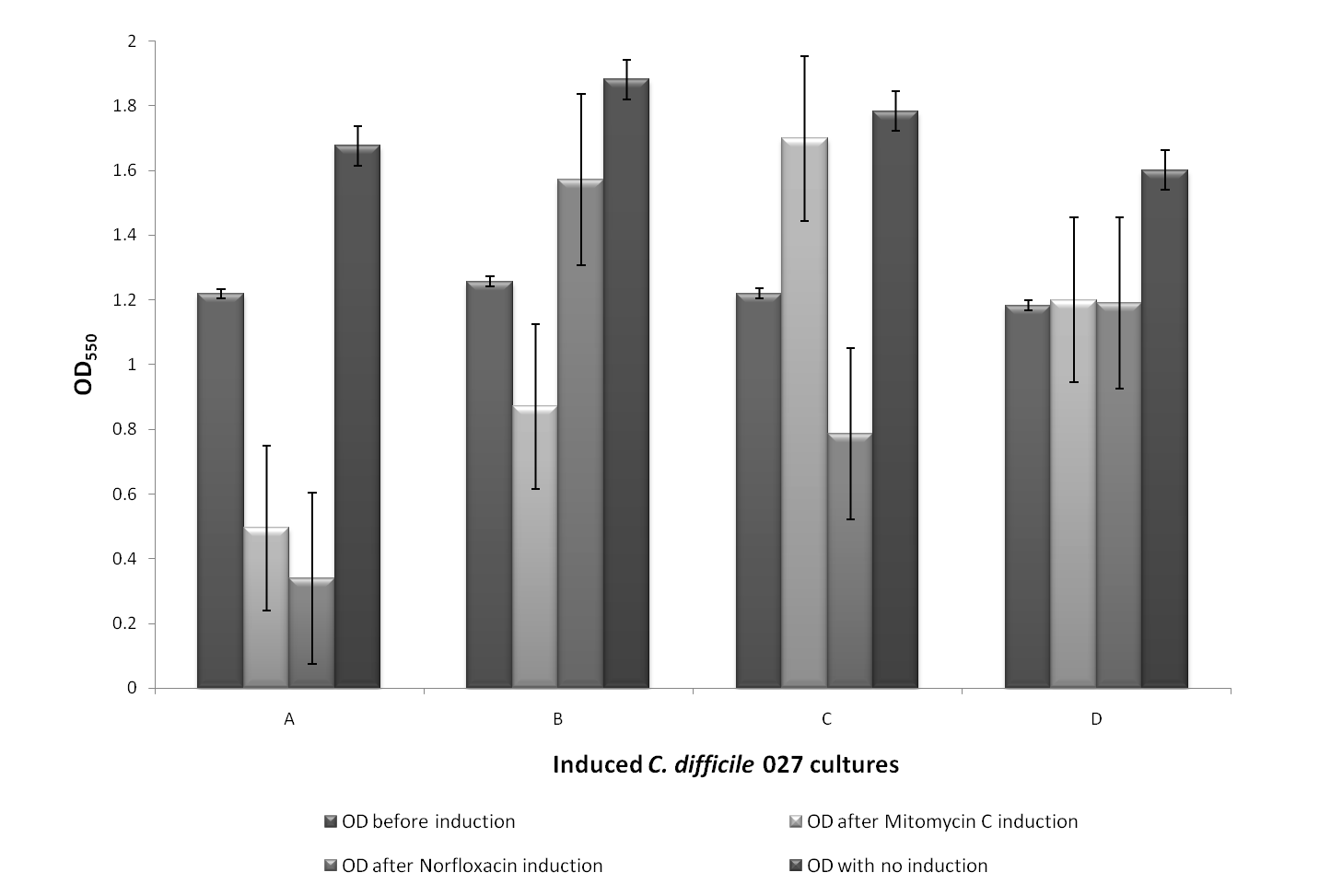

Supplement: Figure S2 — Graph showing different patterns of growth responses of C. difficile cultures during prophage inductions. Overnight broth cultures of C. difficile 027 isolates in BHI at OD550 ∼1.2 were induced with norfloxacin or mitomycin C at final concentration of 3 µg/ml for 24 h. Final OD550 were taken at the end of 24 h. The effect of antibiotics on the growth of bacterial cultures was determined by measuring the OD550 values before and after induction. The growth responses all fitted into one of four patterns (A–D) and this was compared to phage release. I. For pattern A, there was a drop in OD550 with both mitomycin C and norfloxacin inductions. Although this pattern has previously been reported in C. difficile inductions, only 35 of the 91 isolates used in this study showed this profile. Furthermore, from these 35 isolates, 33 were found to harbour intact phages with the remaining two harbouring phage tail-like particles following induction by either antibiotic. II. For pattern B, the OD550 dropped with mitomycin C induction but increased following norfloxacin induction. Twenty-eight of isolates showed this pattern. The TEM analysis showed that there was phage release from 16 of the isolates following mitomycin C and 11 with norfloxacin induction. One isolate was found to contain no phages. III. Pattern C was the opposite of pattern B with OD550 increasing following mitomycin C induction but dropping following norfloxacin induction. Only five isolates showed this pattern. There was phage release from two isolates with mitomycin C and three with norfloxacin induction as confirmed by TEM analysis. IV. For pattern D, the OD550 remained relatively constant following either norfloxacin or mitomycin C induction of the cultures. This pattern was observed in 23 isolates. Only phage tail-like particles were observed with these isolates. In addition to the 24 h observations, 15 isolates belonging to the different patterns were selected and monitored after an induction time of 72 h. [file pone.0037263.s003.tif]

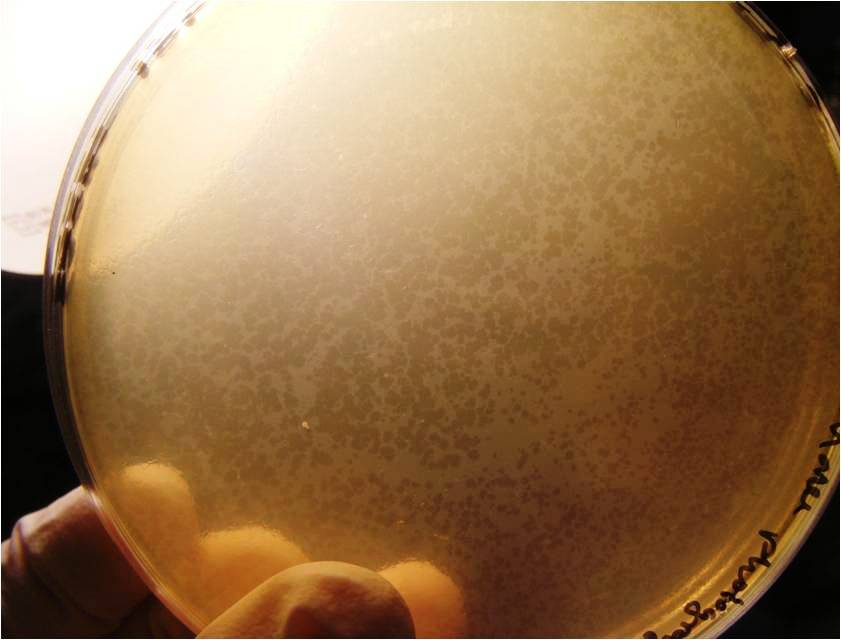

Supplement: Figure S3 — Picture showing plaques of novel myovirus E on lawn of CD630. (TIF) [file pone.0037263.s004.tif]
